# Efomycins K and L From a Termite-Associated *Streptomyces* sp. M56 and Their Putative Biosynthetic Origin

**DOI:** 10.3389/fmicb.2019.01739

**Published:** 2019-08-06

**Authors:** Jonathan L. Klassen, Seoung Rak Lee, Michael Poulsen, Christine Beemelmanns, Ki Hyun Kim

**Affiliations:** ^1^Department of Molecular and Cell Biology, University of Connecticut, Storrs, CT, United States; ^2^School of Pharmacy, Sungkyunkwan University, Suwon, South Korea; ^3^Section for Ecology and Evolution, Department of Biology, University of Copenhagen, Copenhagen, Denmark; ^4^Leibniz Institute for Natural Product Research and Infection Biology e.V., Hans-Knöll-Institute (HKI), Jena, Germany

**Keywords:** elaiophylin, efomycin K, efomycin L, termite-associated bacteria, gene cluster, *ela* cluster

## Abstract

Two new elaiophylin derivatives, efomycins K (**1**) and L (**2**), and five known elaiophylin derivatives (**3–7**) were isolated from the termite-associated *Streptomyces* sp. M56. The structures were determined by 1D and 2D NMR and HR-ESIMS analyses and comparative CD spectroscopy. The putative gene cluster responsible for the production of the elaiophylin and efomycin derivatives was identified based on significant homology to related clusters. Phylogenetic analysis of gene cluster domains was used to provide a biosynthetic rational for these new derivatives and to demonstrate how a single biosynthetic pathway can produce diverse structures.

## Introduction

**FIGURE 1:**
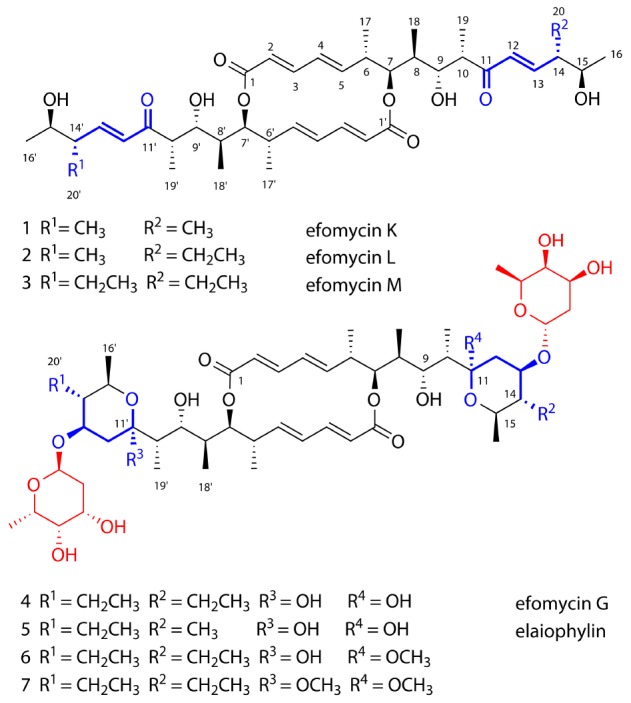
Structures of isolated efomycin and elaiophylin derivatives (**1**–**7**).

Fungus-growing insects, which include several ant, beetle, and termites species (Beemelmanns et al., 2016), host diverse symbiotic organisms within their intestines and surroundings to protect the colony against predatory or pathogenic species (Engl and Kaltenpoth, 2018; Scherlach and Hertweck, 2018). Recent developments in OMICs-based technologies have revolutionized the ways we identify microbial natural products that mediate the interactions between insects and microbes (Wilkinson and Micklefield, 2007; Kuhlisch and Pohnert, 2015; Shendure et al., 2017; Palazzotto and Weber, 2018). Intense chemical investigation has led to the identification of numerous new chemical scaffolds and potential new antibiotics within a short period of time (Berasategui et al., 2016). As an example, our recent metabolomic and genomic studies on microbial symbionts of fungus-growing termites have led to identification of several new secondary metabolites (Kurtböke et al., 2015; Genilloud, 2017; Benndorf et al., 2018). These include new isoflavonoid glycosides (termisoflavones A-C) (Kang et al., 2016), 20-membered glycosylated and antifungal polyketide macrolactams (macrotermycins A-D) (Beemelmanns et al., 2017), the PKS-derived geldanamycin analog natalamycin A, all from the highly antifungal strain *Streptomyces* sp. M56 (Kim et al., 2014), and the antibacterial prerubterolones and rubterolones from *Actinomadura* sp. RB29 (Guo et al., 2017, 2018). As part of these explorations, we were particularly intrigued by the exceptionally high antifungal activity of *Streptomyces* sp. M56 against both the termite mutualistic cultivar (*Termitomyces* spp.) and competitors/antagonists of this cultivar (*Pseudoxylaria* spp.). Our early investigations showed that geldanamycin and new derivatives thereof were partially responsible for the observed antifungal activities (Kim et al., 2014). The structures of the remaining active metabolites remained enigmatic. We therefore revisited these highly antifungal metabolite extracts and conducted detailed activity- and NMR-guided fractionations (Romero et al., 2015). These intensified efforts led to the isolation and characterization of two new elaiophylin derivatives, carrying an unsaturated enone moiety, which we named efomycins K (**1**) and L (**2**), in addition to the known derivative efomycin M (**3**) (Figure 1), a potent and specific inhibitor of selectin (Schön et al., 2002; Supong et al., 2016). Further studies led to the isolation of five known and structurally-related hemiketal derivatives, including efomycin G (**4**) (Frobel et al., 1990), elaiophylin (**5**) (Kaiser and Keller-Schierlein, 1981; Ritzau et al., 1998; Wu et al., 2013), 11-*O*-methylelaiophylin (**6**) (Wu et al., 2013), and 11,11’-*O*-dimethylelaiophylin (**7**) (Kretschmer et al., 1985; Pimentel-Elardo et al., 2015). Due to the intriguing co-production of unsaturated enones (**1**–**3**) and corresponding hemiketals (**4**–**7**), and the increasing interest in the evolution of gene clusters distributed amongst different genera of Actinomycetales, we analyzed the biosynthetic origin of elaiophylins in *Streptomyces* in greater detail.

## Materials and Methods

### General Experimental Procedures

Optical rotations were obtained using a Perkin-Elmer 241 polarimeter. IR spectra were recorded on a Bruker Alpha-P FT-IR spectrometer. UV spectra were acquired on an Amersham Biosciences Ultrospec 5300 Pro Spectrophotometer. CD spectra were measured on a Jasco J-715 spectropolarimeter. HR-ESI mass spectra were recorded on a Waters Micromass Q-ToF Ultima ESI-TOF mass spectrometer at the University of Illinois Urbana-Champaign School of Chemical Sciences Mass Spectrometry Laboratory. LC/MS analysis was performed on an Agilent 1200 Series HPLC system equipped with a diode array detector and a 6130 Series ESI mass spectrometer using an analytical Phenomenex Luna C18 column (5 μm, 4.6 mm × 100 mm). All NMR experiments were carried out on a Varian INOVA 600 MHz NMR spectrometer equipped with an indirect detection probe. HPLC purification was carried out on an Agilent 1100 or 1200 Series HPLC system (Agilent Technologies) equipped with a photo diode array detector. C18 Waters Sep-Pak cartridges were used for column chromatography. Merck precoated silica gel F254 plates and reverse-phase (RP)-18 F254s plates were used for TLC. Spots were detected on TLC under UV light or by heating after spraying with anisaldehyde-sulphuric acid.

### Sequencing and Species Identification

A region of the 16S rDNA gene was amplified for phylogenetic analysis with general primers [27F and 1492R] using standard DNA extraction and PCR protocols (Benndorf et al., 2018). A nucleotide BLAST search of the partial 16S rRNA sequence of *Streptomyces* sp. M56 revealed a 100% identity match with the partial 16S rRNA sequence of *Streptomyces malaysiensis* strain 1160 GenBank accession number HQ607429.1.

Genomic DNA was extracted from a 5-day-old culture of M56 grown in ISP-2 broth at 30 °C with shaking at 200 rpm. Whole-genome sequencing was performed at the Duke University Center for genomic and computational biology (GCB) using PacBio SMRT sequencing technology (PacBio Sequel) and an 8- to 11-kb insert library prepared from M56 gDNA. Reads were assembled to a single unitig using the hierarchical genome assembly process, and low-quality nucleotides were removed manually from the unitig termini. This *de novo* genome assembly was polished once by mapping the reads to the assembled contigs using Quiver, as implemented in the RS_Resequencing.1 protocol in the SMRTportal v 2.3.0.140893. Further rounds of Quiver polishing did not yield any further SNP or indel corrections. The final assembly was annotated using Prokka v1.11 (Seemann, 2014) and deposited in the NCBI database as accession number CP025018.1. The assembled and annotated *Streptomyces* sp. M56 genome is composed of a single contig 1,1742,376 bp long with a %GC of 71.0. Overall, the sequence coverage is 94× with a mean nucleotide quality value of 48.4, which translates to a 0.0014% expected error rate.

Strain M56 shares 98.64% ANI^1^ with the previously described species *S. malaysiensis* DSM4137 (note that *S. malaysiensis* DSM4137 was formerly classified as a strain of *Streptomyces violaceusniger*). Although *S. malaysiensis* DSM4137 is not a type strain of this genus (or listed in the current DSM database), its 16S rRNA gene is 99% identical (NCBI BLASTn vs. nr May 5/18) to that of the type strain *S. malaysiensis* ATB-11 (NCBI accession NR_114497), and we therefore consider all three isolates to be the same species.

### Gene Cluster Analysis

The elaiophylin BGC in *Streptomyces* sp. M56 was identified based on its biosynthetic logic and high homology to the previously studied elaiophylin BGCs in *S. malaysiensis* DSM4137 (*S. violaceusniger*) and “*Streptomyces autolyticus*” CGMCC0516. All genes present in the reference clusters were also present in *Streptomyces* sp. M56, including those for regulation, export, precursor and sugar biosynthesis, PKS, and tailoring enzymes (Supplementary Figures S2–S4 and Supplementary Tables S1, S2). The first PKS gene in the *S. malaysiensis* Ela BGC was fragmented in the genome annotation but is reported as a single intact gene here (Kim et al., 2014). The core Ela BGC comprises 5 PKS genes that include 8 PKS modules in total, as well as a separate and free-standing TE domain ElaF following the final fifth PKS module (Supplementary Figure S1 and Supplementary Table S1).

### Sequence Alignments

Sequence alignment of ketoreductase domains from this study with the signature sequences reported by Keatinge-Clay (2007). Lid regions of the KR domains are not shown. Alignment was generated using MUSCLE (Edgar, 2004).

### Cultivation and Extraction Procedure of *Streptomyces* sp. M56

*Streptomyces* sp. M56 was grown on 50 ISP-2 (BD Difco^TM^ ISP-2 medium and 1.5% Agar-Agar) agar plates (14 cm diameter) for 10 days at 30°C (Supplementary Figures S5, S6). The agar was then cut into squares, consolidated, and soaked overnight in *i*PrOH. The *i*PrOH phase was filtered, and the solvent was concentrated under vacuum to afford a crude extract, which was dissolved in 80% MeOH/H_2_O (200 mL). The extract was loaded onto an activated pre-packed C18 Sep-Pak cartridge (10 g, Waters) equilibrated with 20% MeOH/H_2_O. The charged column was then washed with two column volumes of 20% MeOH/H_2_O to remove very polar compounds, followed by step gradient elution with two column volumes of each of the following solvent mixtures: 40% MeOH/H_2_O, 60% MeOH/H_2_O, 80% MeOH/H_2_O, 100% MeOH, and 100% acetone. Each fraction was tested for antifungal and antibacterial activities in triplicate against standardized bacterial and yeast strains from the American Type Culture Collection [*Bacillus subtilis* (ATCC 6633), *Escherichia coli* (ATCC 25922), and *Saccharomyces cerevisiae* (ATCC 9763)]. Fractions eluted with 80% MeOH/H_2_O and 100% MeOH exhibited a clear zone of inhibition in disc diffusion assays, with an minimal inhibitory concentration (MIC) of 35 ± 5 μg/mL for *B. subtilis* and an minimum fungicidal concentration (MFC) of 45 ± 5 μg/mL for *S. cerevisiae*. These fractions were subsequently analyzed by LC-MS, which indicated a complex mixture of secondary metabolites. The fractions eluted with 80% MeOH/H_2_O and 100% MeOH were consolidated and subsequently purified by preparative reverse-phase HPLC (Agilent 1100 Series HPLC system, C18 column, Phenomenex Luna, 250 mm × 21.2 mm, 5 μm) with a flow rate of 10 mL/min using a linear gradient from 30% MeOH/H_2_O to 100% MeOH for 30 min, 100% MeOH for the next 10 min, 30% MeOH/H_2_O within 1 min, and further isocratic elution for 9 min. Fractions were collected for every minute from 6 to 40 min to produce 34 fractions. These fractions were tested in an assay against *B. subtilis* and *S. cerevisiae*. Fractions 20–28 exhibited moderate to strong activity against *B. subtilis* and *S. cerevisiae*. Among the active fractions, fraction 25 was purified by semi-preparative reverse-phase HPLC (55% MeCN/H_2_O + 0.1% formic acid) using a phenyl-hexyl column (Phenomenex Luna, 250 mm × 10.0 mm, 5 μm, flow rate: 2 mL/min) to yield compound **1** (0.6 mg, *t*_R_ = 17.4 min) and the previously reported geldanamycin and derivatives (Kim et al., 2014). Fraction 26 was purified by semi-preparative reverse-phase HPLC (55% MeCN/H_2_O + 0.1% formic acid) using the phenyl-hexyl column (flow rate: 2 mL/min) to furnish compounds **2** (0.7 mg, *t*_R_ = 20.4 min) and **3** (0.9 mg, *t*_R_ = 24.0 min). Fraction 27 was separated by purification of the semi-preparative reverse-phase HPLC using a gradient program [water + 0.1% formic acid (A), acetonitrile (B): 0–33 min: 45% B; 33–34 min: 45%→100% B; 34–50 min: 100% B] with a C8 column (Phenomenex Luna, 250 × 10.0 mm, 5 μm, flow rate: 2 mL/min) to give compounds **5** (1.5 mg, *t*_R_ = 21.6 min), **4** (1.4 mg, *t*_R_ = 27.8 min), **6** (0.5 mg, *t*_R_ = 42.1 min), and **7** (0.4 mg, *t*_R_ = 46.1 min) (Supplementary Figures S7–S21).

#### Efomycin K (1)

Amorphous powder; ${\left[\alpha \right]}_{D}^{25}$ + 17.4 (*c* 0.03, MeOH); IR (KBr) ν_max_ 3421, 2950, 2830, 1707, 1645, 1600, 1483, 1380, 1309, 1182, 1023, 920 cm^-1^; UV (MeOH) λ_max_ (log *𝜀*) 252 (4.8) nm; CD (MeOH) 210.0 (Δ*𝜀* -15.8), 250.5 (Δ*𝜀* -40.5), 279.0 (Δ*𝜀* +32.0) nm; ^1^H (CD_3_OD, 600 MHz) and ^13^C NMR (CD_3_OD, 150 MHz) data, see Table 1; positive HR-ESIMS *m/z* 701.4251 [M + H]^+^ (calcd. for C_40_H_61_O_10_, 701.4265).

**Table 1:** ^1^H (600 MHz) and ^13^C NMR (150 HMz) data of efomycin K (**1**) and M (**2**) in CD_3_OD.^a^

Position	1		2	
	*δ*_C_	*δ*_H_ (*J* in Hz)	*δ*_C_	*δ*_H_ (*J* in Hz)
1	169.6, s		169.6, s	
2	122.6, d	5.70, d (15.0)	122.6, d	5.70, d (15.0)
3	146.6, d	6.87, dd (15.0, 11.0)	146.6, d	6.87, dd (15.0, 11.0)
4	132.4, d	6.13, dd (15.0, 11.0)	132.4, d	6.13, dd (15.0, 11.0)
5	145.8, d	5.63, dd (15.0, 10.5)	145.8, d	5.63, dd (15.0, 10.5)
6	42.9, d	2.55, m	42.8, d	2.55, m
7	77.8, d	5.15, br d (11.0)	77.7, d	5.14, br d (9.5)
8	37.9, d	1.93, m	37.9, d	1.93, m
9	73.5, d	3.75, dd (9.5, 3.5)	73.4, d	3.75, dd (9.0, 3.5)
10	47.5, d	3.07, qd (6.5, 3.5)	47.0, d	3.12, qd (7.0, 3.5)
11	204.8, s		204.7, s	
12	130.0, d	6.22, d (15.5)	132.2, d	6.17, d (16.0)
13	151.1, d	6.87, dd (15.5, 4.0)	149.6, d	6.72, dd (16.0, 4.0)
14	45.3, d	2.34, qd (7.0, 5.5)	53.6, d	2.04, m
15	71.4, d	3.70, qd (6.5, 5.5)	70.2, d	3.76, qd (6.5, 5.5)
16	20.9, q	1.11, d (6.5)	21.4, q	1.11, d (6.5)
17	15.6, q	1.02, d (6.5)	15.6, q	1.02, d (6.5)
18	10.0, q	0.99, d (7.0)	10.0, q	1.00, d (6.5)
19	9.2, q	1.09, d (6.5)	9.1, q	1.09, d (7.0)
20	15.8, q	1.05, d (7.0)	24.9, t	1.60, m; 1.43, m
21			12.3, q	0.84, t (7.5)
1’	169.6, s		169.6, s	
2’	122.6, d	5.70, d (15.0)	122.6, d	5.69, d (15.0)
3’	146.6, d	6.87, dd (15.0, 11.0)	146.6, d	6.87, dd (15.0, 11.0)
4’	132.4, d	6.13, dd (15.0, 11.0)	132.4, d	6.13, dd (15.0, 11.0)
5’	145.8, d	5.63, dd (15.0, 10.5)	145.8, d	5.63, dd (15.0, 10.5)
6’	42.9, d	2.55, m	42.8, d	2.55, m
7’	77.8, d	5.15, br d (11.0)	77.7, d	5.14, br d (9.5)
8’	37.9, d	1.93, m	37.9, d	1.93, m
9’	73.5, d	3.75, dd (9.5, 3.5)	73.4, d	3.75, dd (9.0, 3.5)
10’	47.5, d	3.07, qd (6.5, 3.5)	47.4, d	3.07, qd (7.0, 3.5)
11’	204.8, s		204.7, s	
12’	130.0, d	6.22, d (15.5)	130.0, d	6.22, d (16.0)
13’	151.1, d	6.87, dd (15.5, 4.0)	151.1, d	6.86, dd (16.0, 4.0)
14’	45.3, d	2.34, qd (7.0, 5.5)	45.2, d	2.34, qd (6.5, 5.5)
15’	71.4, d	3.70, qd (6.5, 5.5)	71.4, d	3.70, qd (6.5, 5.5)
16’	20.9, q	1.11, d (6.5)	21.0, q	1.12, d (6.5)
17’	15.6, q	1.02, d (6.5)	15.6, q	1.02, d (6.5)
18’	10.0, q	0.99, d (7.0)	10.0, q	0.99, d (6.5)
19’	9.2, q	1.09, d (6.5)	9.1, q	1.09, d (7.0)
20’	15.8, q	1.05, d (7.0)	15.8, q	1.05, d (6.5)

*^*a*^Coupling constants (in parentheses) are in Hz; ^*13*^C NMR data were assigned based on HSQC and HMBC experiments (Supplementary Figures S7–S21).*

#### Efomycin L (2)

Amorphous powder; ${\left[\alpha \right]}_{D}^{25}$ + 18.0 (*c* 0.04, MeOH); IR (KBr) ν_max_ 3420, 2950, 2836, 1706, 1645, 1610, 1480, 1384, 1303, 1182, 1019, 920 cm^-1^; UV (MeOH) λ_max_ (log *𝜀*) 252 (4.8) nm; CD (MeOH) 211.0 (Δ*𝜀* -13.8), 249.5 (Δ*𝜀* -44.6), 279.0 (Δ*𝜀* +31.9) nm; ^1^H (CD_3_OD, 600 MHz) and ^13^C NMR (CD_3_OD, 150 MHz) data, see Table 1; positive HR-ESIMS *m/z* 715.4424 [M + H]^+^ (calcd. for C_41_H_63_O_10_, 715.4421).

## Results and Discussion

### Isolation and Structure Elucidation of Efomycin/Elaiophylin Congeners

*Streptomyces* sp. M56 was grown on ISP-2 agar plates for 14 d and mycelium covered plates were extracted using *iso*-propanol (*i*PrOH). The resulting crude extract was subjected to C18 solid phase (SP) extraction using a 10% step elution gradient from 20% MeOH/H_2_O to 100% MeOH (one column volume per step). Resulting fractions were tested for antimicrobial activity against *S. cerevisiae* using a disk diffusion assay. Fractions eluted with 80% MeOH in H_2_O and 100% MeOH exhibited strong antifungal activity and were subjected to preparative-scale C18 reverse-phase HPLC. In depth ^1^H NMR and HRMS-analyses of these active RP-HPLC fractions revealed both previously reported geldanamycin derivatives (Kim et al., 2014) and a group of highly similar unsaturated polyketide-derived compounds with characteristic UV absorption spectra. Subsequent semi-preparative reverse-phase HPLC separation of antifungal fractions accompanied by NMR analysis yielded seven efomycin/elaiophylin congeners, including two previously unreported structural derivatives **1** and **2**.

**FIGURE 2:**
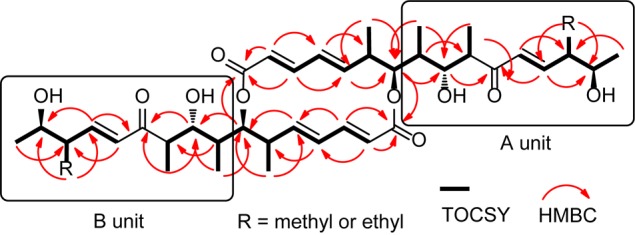
Key TOCSY and HMBC correlations of **1** and **2**.

For compound **1**, HR-ESI-MS analysis provided the pseudomolecular ion of 701.4251 [M+H]^+^, consistent with a molecular formula of C_40_H_60_O_10_. ^1^H NMR analysis (Table 1) indicated the presence of 10 methyl signals and 12 olefinic and six oxygenated methine protons. The observed ^1^H NMR chemical shifts were very similar to those of compounds **2** and **3** (*vide infra*), with the major difference of **1** being the absence of a triplet (CH_3_) proton signal at *δ*_H_ 0.84. The planar structure of **1** was determined by 2D NMR analysis (^1^H-^1^H COSY, TOCSY, HSQC, and HMBC). Distinct HMBC correlations along with ^1^H-^1^H COSY and TOCSY correlations revealed a 16-membered macrolide ring structure and demonstrated a connection between units A and B, respectively (Figure 2 and Supplementary Figure S7). Comparison of the NMR data with those of efomycin M (**3**) implied that compound **1** carries two methyl groups at C-14/C-14’, instead of ethyl groups, as in the case of **3** (Schön et al., 2002).

HR-ESI-MS analysis of compound (**2**) provided the unfamiliar molecular formula of C_41_H_62_O_10_ (715.4424 [M + H]^+^). Again, ^1^H NMR data (Table 1) indicated the presence of 10 methyl signals and 12 olefinic and six oxygenated methine protons. The ^13^C NMR results assigned by HSQC and HMBC spectra displayed the presence of 41 carbon signals, which were classified into 10 methyls, one methylene, 26 methines including 12 olefinic carbons, and 4 quaternary carbonyl carbons. Comparison of the NMR data with those of compounds **1** and **3** implied that compound **2** was highly similar but asymmetric. The planar structure of **2** was again determined by 2D-NMR analysis (^1^H-^1^H COSY, TOCSY, HSQC, and HMBC) and comparison of chemical shifts (Figure 2). Distinct HMBC correlations of H_3_-21/C-14 and H_3_-21/C-20 to C-14 revealed that compound **2** possessed one ethyl group linked to C-14 rather than a methyl group as found in compound **1** and was therefore named efomycin L. The absolute stereochemistry of efomycins **1**–**3** was deduced from highly similar NMR patterns, including coupling constants, the almost identical ECD spectral data [**1**: 211.0 (Δ*𝜀* -13.8), 249.5 (Δ*𝜀* -44.6), 279.0 (Δ*𝜀* +31.9), **2**: 210.0 (Δ*𝜀* -15.8), 250.5 (Δ*𝜀* -40.5), 279.0 (Δ*𝜀* +32.0), **3**: 208.5 (Δ*𝜀* -11.7), 249.5 (Δ*𝜀* -47.8), 278.0 (Δ*𝜀* +30.8) and **4**: 216.0 (Δ*𝜀* -23.3), 249.0 (Δ*𝜀* -43.8), 279.0 (Δ*𝜀* +33.9)], and the biosynthetic origin (*vide infra*). Furthermore, structurally closely related metabolite efomycin G (**4**) (Frobel et al., 1990), elaiophylin (**5**) (Arai, 1960), 11-*O*-methylelaiophylin (**6**) (Wu et al., 2013), and 11,11’-*O*-dimethylelaiophylin (**7**) (Ritzau et al., 1998) were isolated.

Elaiophylins are glycosylated C2 symmetric 16-membered macrolides that are derived from two linear polyketide chains. Most structural variations of elaiophylins vary by the absence or presence of the glycosylated dihydroxypyrane moiety and different substitution patterns at C-11/C-11′ (hydroxy or methoxy groups) (Wienrich et al., 2006; Sheng et al., 2015). A highly intriguing feature in this study is the co-occurrence of efomycins (**1**–**3**) containing an unsaturated enone moiety and structurally derived elaiophylins (**4**–**7**), which are formed via a stereospecific intra-molecular hemiacetal process. Similar intra-molecular hemiacetal can be found in the PKS-derived C2 symmetric congoblatin (Zhou et al., 2015b), vermiculin (Proksa and Fuska, 2000), and plecomacrolides (Dai et al., 2005), a large family of 16- or 18-membered macrolactones. Intriguingly, the distantly related polyketide pair oxohygrolidin and hygrolidin show the same structural elements, an unsaturated enone and the corresponding hemiketals, respectively.

Elaiophylins also exhibit remarkable pharmacological properties such as antimicrobial, anticancer, immunosuppressant, and antiviral activity (Ritzau et al., 1998; Doseung et al., 2011; Lim et al., 2018). Similar to bafilomycins, the antibacterial elaiophylins have been shown to enhance antifungal activity in combination with co-produced rapamycin (Fang et al., 2000). Furthermore, efomycin M was found to be non-toxic and showed selective inhibitory effects on selectin-mediated leukocyte-endothelial adhesion *in vitro* (Schön et al., 2002). Thus, we speculate that in combination with other produced metabolites, such as the previously identified geldanamycin analogs (Kim et al., 2014), elaiophylins and/or efomycins could be responsible for the high antifungal activity observed in the herein investigated *Streptomyces* sp. M56 metabolite extracts.

### Identification of the Putative Biosynthetic Gene Cluster of Efomycin/Elaiophylin Congeners

To date, the molecular complexity of these unsaturated macrolactones has hampered synthetic approaches that would enable detailed structure-function analyses and improvement of pharmacological properties. Thus, detailed information about the biosynthetic pathways of efomycins and elaiophylins within the producing organism *Streptomyces* sp. M56 will enable future pathway engineering to increase production titers and to create new structural derivatives with altered potency and activity spectra (Dorsch et al., 2004).

To identify the biosynthetic origin of **1**–**7**, the *Streptomyces* sp. M56 genome was sequenced (NCBI accession number CP025018.1). *De novo* genome assembly was performed using the PacBio SMRTportal with the HGAP protocol, polished using Quiver, and annotated using Prokka v1.11 (Seemann, 2014 Bioinformatics). The assembled and annotated *Streptomyces* sp. M56 genome is composed of a single 1,1742,376-bp single contig with a 71.0% GC content. Strain M56 shares 98.64% ANI^2^ with the previously described species *S. malaysiensis* DSM4137. Similarly, the genome of “*Streptomyces autolyticus*” CGMCC0516 shares 98.68% and 98.64% ANI with strain M56 and *S. malaysiensis*, respectively, indicating that all three strains should be considered as belonging to the same bacterial species.

Secondary metabolite biosynthetic gene clusters (BGCs) were predicted in *Streptomyces* sp. M56 and the genomes of *S. malaysiensis* DSM4137 (NCBI accession CP023992.1) and “*S. autolyticus*” CGMCC0516 (NCBI accession CP019458) using antiSMASH v4.1.0 with default parameters.

**FIGURE 3:**
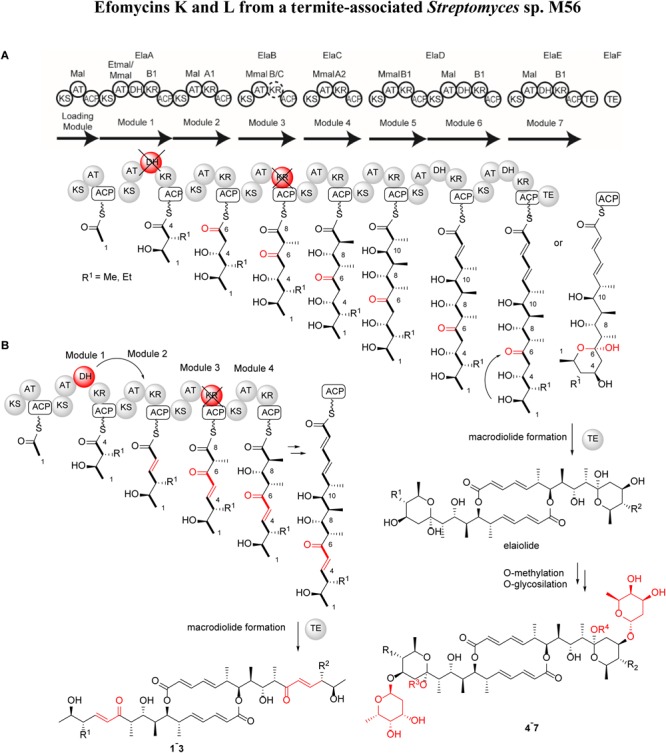
Proposed efomycins (**1–3**) and elaiophylins (**4–7**) PKS assembly lines. Domain composition and specificity of the *ela* BGCs; the dimerization mechanism was adapted from Zhou et al. (2015b). Pathway **(A)** biosynthetic assembly line proposes an inactive ElaA-DH and ElaB-KR domains (colored in red). Pathway **(B)** biosynthetic assembly line proposes a *trans*-acting ElaA-DH and an inactive ElaB-KR domain (colored in red).

Previous studies have shown that the macrolactone core of elaiophylins and related structures is assembled by a type I PKS system following the PKS pattern of collinearity (Gerlitz et al., 1992; Weissman, 2015; Zhou et al., 2015a). The elaiophylin BGC in *Streptomyces* sp. M56 was identified based on its biosynthetic logic and high homology to the previously studied elaiophylin BGCs in *S. malaysiensis* DSM4137 (Supplementary Figure S1 and Supplementary Table S1). All genes present in the reference clusters were also present in *Streptomyces* sp. M56, including those for regulation, export, precursor and sugar biosynthesis, PKS, and tailoring enzymes. The core *ela* cluster consists of five PKS genes that include eight PKS modules in total, as well as a separate and free-standing TE domain ElaF following the final fifth PKS module (Figure 3 and Supplementary Figure S1; Haydock et al., 2004). The substrate specificities of each AT (Supplementary Figure S4) and the direction of ketoreduction of each KR domain (Supplementary Figures S2, S3) were assigned based on phylogenetic relatedness to domains with known specificities and signature amino acid sequences. These domain assignments are consistent and collinear with the elaiophylin/efomycin structures with three important features.

First, it needs to be noted that efomycins (**1**–**3**) and elaiophylins (**4**–**7**) carry either a methyl or ethyl residue at position C-14. Thus, it is conceivable that either a promiscuous AT-domain (ElaA) or an unusually high production titre of ethyl malonate shifts the production towards derivatives carrying an ethyl substituent.

Secondly, module 1 includes a dehydratase, which we propose acts *in trans* as part of Module 2, mediated by ElaB based on dehydration at this latter position and the malonyl unit at this position that is consistent with the AT domain specificity of Module 2 but not of that of Module 1 or 3 (Chen and Du, 2016).

Thirdly, the Module 3 KR domain shows a hybrid amino acid signature that contains the “LDD” motif typical of B-type KR domains but lacks the conserved “N” residue found in both A- and B-type KR domains (Supplementary Table S2). Thus, the Module 3 KR domain likely functions as a typical C-type domain that lacks ketoreductase activity (Keatinge-Clay, 2007; Zheng and Keatinge-Clay, 2011). We therefore annotated this domain as a hybrid B/C KR type and assumed that it is not active, consistent with the presence of a non-reduced keto group at C-6. Examination of the sequence of the encoded TE domain showed it to be very similar to those catalyzing intramolecular lactonization (type I TE) (Hari et al., 2014). At this stage, it cannot be excluded that both TE domains act synergistically to catalyze the two acylation and deacylation steps during macrodiolide formation; and the exact mode of action of the domains remains to be elucidated.

Overall, this arrangement generates two possible PKS-products, one of which carries β-hydroxy ketone functionality (pathway A, Figure 3) and allows for downstream hemiketal formation, as seen in formation of compounds **4**–**7**, and modification by addition of 2-deoxy-l-fucose at C-13 by ElaG (Weissman, 2015). In contrast, formation of the more rigid unsaturated enone moiety via pathway B most likely prevents the intramolecular hemiacetal formation observed for compounds **1**–**3**.

## Conclusion

In conclusion, our activity- and genome-guided analysis of termite-associated Actinobacteria led to the isolation of two new efomycin derivatives in *Streptomyces* sp. M56. The simultaneous production of antimicrobial geldanamycins, efomycins, and elaiophylins and their likely synergistic bioactivities are presumably the reasons for the observed high antifungal activity of *Streptomyces* sp. M56 and future studies will be directed at understanding the regulation of their production and bioactivities. The increasing interest in evolution of gene clusters in Actinobacteria led us to pursue a detailed *in silico* biosynthetic pathway analysis of the putative *ela* cluster. The identified putative biosynthetic gene cluster harbors a DH that presumably needs to act in *trans* to produce both enones (efomycins **1**–**3**) and hemiacetals (elaiophylins **4**–**7**). Future molecular biological analyses will sheet light into the exceptions to the co-linearity principle and aid in the understanding of the complex control of secondary metabolism in *Streptomyces* sp. M56 and beyond.

## Data Availability

Genomic datasets generated for this study can be found in the Genbank Accession number: KJ511242.

## Author Contributions

CB and KK conceived the study. All authors conducted the experiments. JK and CB contributed to the bioinformatics analysis. JK, CB, and KK wrote the manuscript with help from SL and MP.

## Conflict of Interest Statement

The authors declare that the research was conducted in the absence of any commercial or financial relationships that could be construed as a potential conflict of interest.

## Footnotes

1 http://enve-omics.ce.gatech.edu/ani/

2 http://enve-omics.ce.gatech.edu/ani/
