# Supplementary material for: Efomycins K and L From a Termite-Associated Streptomyces sp. M56 and Their Putative Biosynthetic Origin
Source: Front Microbiol. 2019 Aug 6;10:1739. doi: 10.3389/fmicb.2019.01739 (PMC6691879; doi:10.3389/fmicb.2019.01739)
Supplement: Supplementary file 1 [file Data_Sheet_1.PDF]

Supplementary Material

Efomycins K and L from a termite-associated *Streptomyces* sp. M56 and their putative biosynthetic origin

Jonathan L. Klassen[1], Seoung Rak Lee[2], Michael Poulsen[3], Christine Beemelmanns[4]*, and Ki Hyun Kim[2]*

[1]Department of Molecular & Cell Biology, University of Connecticut, Storrs, CT, 06269, USA

[2]School of Pharmacy, Sungkyunkwan University, Suwon, 16419, Republic of Korea

[3]Section for Ecology and Evolution, Department of Biology University of Copenhagen, 2100 Copenhagen East, Denmark

[4]Leibniz Institute for Natural Product Research and Infection Biology e.V., Hans-Knöll-Institute (HKI), Beutenbergstrasse 11a, 07745 Jena, Germany

Contents

1. General Experimental Procedures 3
2. Gene Cluster Analysis 4
3. Metabolomic Analysis 10
4. Structure Analysis and Analytical Data 11

List of Tables

Table S1. Homology between proteins encoded by the *Streptomyces* sp. M56 *ela* gene cluster and their homolog s in *S. malaysiensis* DSM4137 (*Sv.*) and "*S. autolyticus*" CGMCC0516 ("*Sa*"). 5

Table S2. KR domain specificities for *ela* gene clusters 7

List of Figures

Figure S1. Phylogenetic analysis based on near full-length 16S rRNA sequences of isolated Actinobacteria (figure adapted from Benndorf et al. 2018) 4

Figure S1. Comparison of *ela* BGCs. Coloured genes are homologous and named following the "*S. autolyticus*" nomenclature and domain composition and specificity of the *ela* BGCs. 6

Figure S2. Phylogenetic analysis of KR domains of type 1 PKS-related biosynthetic gene clusters found in M56 (*ela*, *oxh*, *baf*/*bfm*, and *unk*). Protein sequences were aligned using MUSCLE v3.8.31 using default parameters, and approximate maximum likelihood trees were constructed using RAxMLv8.0.26 with automatic model select ion and 1000 bootstrap pseudo-replicates. Only bootstrap values ≥ 60% are shown. The tree was rooted to an arb itrarily long branch for clarity, and letters to the right of each set of orthologous domains indicate their domain s pecificity. 8

Figure S3. Phylogenetic analysis AT domain type 1 PKS-related biosynthetic gene clusters found in M56 (*ela*, *o xh*, *baf*/*bfm*, and *unk*). Protein sequences were aligned using MUSCLE v3.8.31 using default parameters, and ap proximate maximum likelihood trees were constructed using RAxMLv8.0.26 using automatic model selection a nd 1000 bootstrap pseudo-replicates. Only bootstrap values ≥ 60% are shown. The tree was rooted to an arbitrari ly long branch for clarity, and the domain specificities assigned previously to the Baf/Bfm AT domains are indic ated beside each clade. 9

Figure S4. Adapted LC-MS diagram (254 nm) of metabolite extracts from *Streptomyces* sp. M56 grown on ISP-2 agar plates after 9 d (blue), 10 d (red), 12 d (green), and 14 d (pink) 10

Figure S5. Preparative HPLC chromatogram (254 nm) of metabolite extracts from *Streptomyces* sp. M56 grown on ISP-2 agar plates. 10

Figure S6. Key ^{1}H-^{1}H COSY and TOCSY (blue lines) and HMBC (red arrows) correlations for compounds **1** and **2**. 11

Figure S7. HR-ESI-MS data of **1**. 12

Figure S8. ^{1}H NMR spectrum of **1.** 12

Figure S9. ^{1}H-^{1}H COSY spectrum of **1** 13

Figure S10. TOCSY spectrum of **1** 13

Figure S11. HSQC spectrum of **1** 14

Figure S12. HMBC spectrum of **1** 14

Figure S13. ECD spectrum of **1** 15

Figure S14. HR-ESI-MS data of **2.** 16

Figure S15. ^{1}H NMR spectrum of **2.** 16

Figure S16. ^{1}H-^{1}H COSY spectrum of **2.** 17

Figure S17. TOCSY spectrum of **2.** 17

Figure S18. HSQC spectrum of **2.** 18

Figure S19. HMBC spectrum of **2** 18

Figure S20. ECD spectrum of **2.** 19

1. General Experimental Procedures

Broth (per 1 L)

ISP-2: 0.4% yeast extract, 1.0% malt extract, 0.4% glucose, pH 7.2

LB-Miller: 1.0% tryptone, 5.0% yeast extract, 1.0% NaCl, pH 7.0

PD: 0.4% potato starch infusion, 2.0% dextrose, pH 5.1

YM: 0.5% peptic digest of animal tissue, 0.3% yeast extract, 0.3% malt extract, pH 6.2

YP: 1.0% yeast, 2.0% peptone, 1.0% glucose, pH 7.0

Agar (per 1 L)

ISP-2: 0.4% yeast extract, 1.0% malt extract, 0.4% glucose, 1.5% agar, pH 7.2

LB-Miller: 1.0% tryptone, 5.0% yeast extract, 1.0% NaCl, 1.5% agar, pH 7.0

2. Gene Cluster Analysis

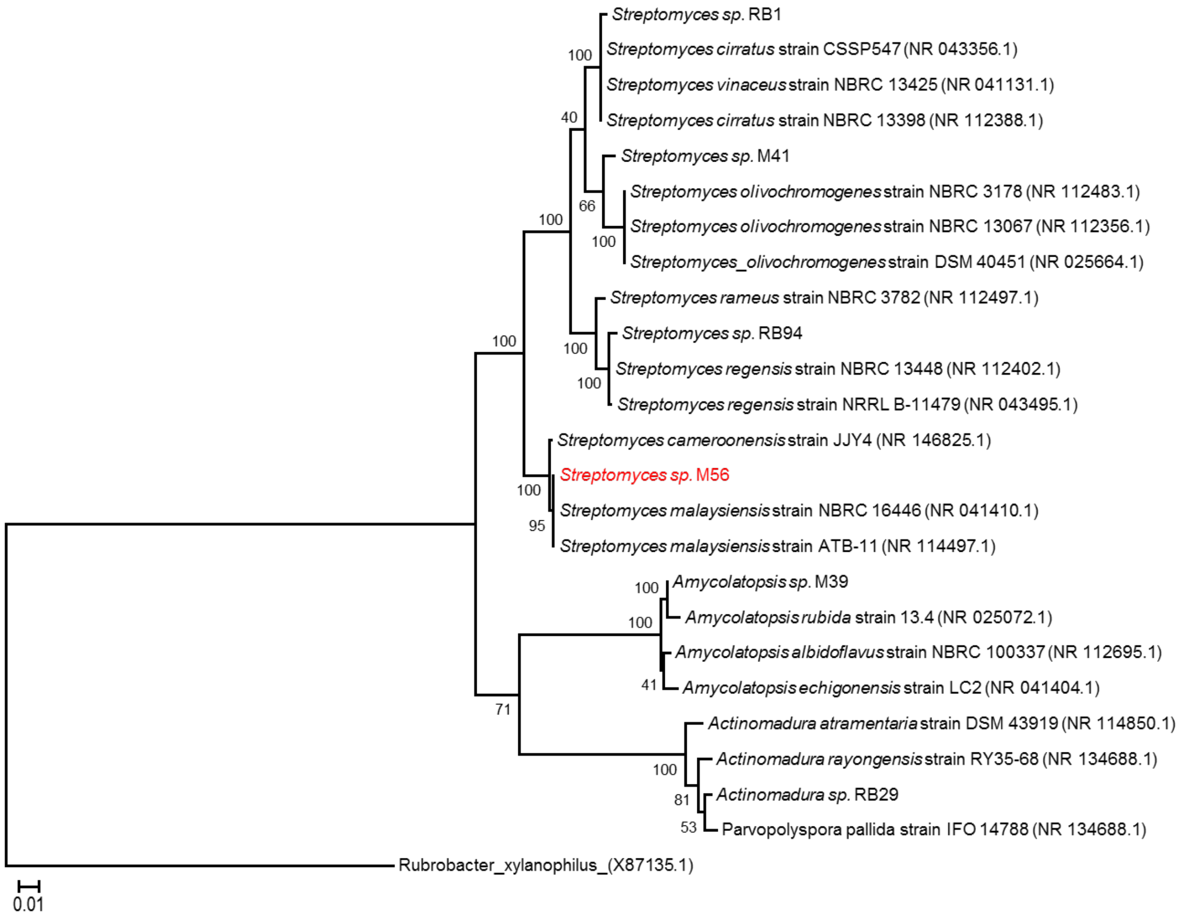

Figure S1. Phylogenetic analysis based on near full-length 16S rRNA sequences of isolated Actinobacteria (figure adapted from Benndorf et al. 2018)

Benndorf, R., Guo, H., Sommerwerk, E., Weigel, C., Garcia-Altares, M., Martin, K., et al. (2018). Natural products from Actinobacteria associated with fungus-growing termites. *Antibiotics* 7, pii: E83. doi: 10.3390/antibiotics7030083.

Table S1. Homology between proteins encoded by the *Streptomyces* sp. M56 *ela* gene cluster and their homologs in *S. malaysiensis* DSM4137 (*Sv.*) and "*S. autolyticus*" CGMCC0516 ("*Sa*").

M56 Gene	NCBI Accession	Annotation	*Sv.* Gene	% Identity/ % Aligned	"*Sa*" Gene	% Identity/ % Aligned
Ela6	AUA09473.1	Hypothetical	Ela6	99/100		
Ela5	AUA09472.1	Hypothetical	Ela5	99/100	Ela5	99/100
Ela4	AUA09471.1	Hydroxybutyryl CoA dehydrogenase	Ela4	100/100	Ela4	99/100
Ela3	AUA09470.1	LuxR family regulator	Ela3	100/85	Ela3	100/97
Ela2	AUA09469.1	TDP glucose synthase	Ela2	100/100	Ela2	100/100
Ela1	AUA09468.1	TDP glucose 4,6 dehydratase	Ela1	99/100	Ela1	100/100
ElaA	AUA09467.1	Type I PKS	ElaA	97/100	ElaA*	99/45
ElaB	AUA09466.1	Type I PKS	ElaB	98/100	ElaB	97/100
ElaC	AUA09465.1	Type I PKS	ElaC	98/100	ElaC	98/100
ElaD	AUA09464.1	Type I PKS	ElaD	99/100	ElaD	99/100
ElaE	AUA09463.1	Type I PKS	ElaE	98/100	ElaE	99/100
ElaF	AUA09462.1	Thioesterase	Ela1*	100/100	ElaF	100/100
ElaG	AUA09461.1	Gylcosyltransferase	Ela2*	99/100	ElaG	99/100
ElaH	AUA09460.1	4-Keto-6-deoxyglucose 3,5-epimerase	Ela3*	98/100	ElaH	97/100
ElaI	AUA09459.1	Exporter (membrane domain)	Ela4*	100/100	ElaI	100/100
ElaJ	AUA09458.1	Exporter (ATPase domain)	Ela5*	100/100	ElaJ	100/100
ElaK	AUA09457.1	Two-component regulator (sensor/kinase domain)	Ela6*	99/100	ElaK	100/100
ElaL	AUA09456.1	Two-component regulator (effector domain)	Ela7*	100/100	ElaL	100/84
ElaM	AUA09455.1	NDP-hexose 4 ketoreductase	Ela8*	100/73	ElaM	99/100
ElaN	AUA09454.1	NDP-hexose 2,3 enoyl reductase	Ela9*	99/100	ElaN	99/100
ElaO	AUA09453.1	NDP-hexose 2,3 dehydratase	Ela10*	100/100	ElaO	100/100
ElaP	AUA09452.1	Crotonyl CoA reductase	Ela11*	99/100	ElaP	100/100
ElaQ	AUA09451.1	Hypothetical	Ela12*	99/100	ElaQ	99/100

*ElaA was split into two genes in the "*S. autolyticus* CGMCC0516 gene cluster, NCBI accession numbers AQA15540.1 and AQA15441.1. This percentage represents the sum of these two sequences that aligned to the M56 ElaA sequence.

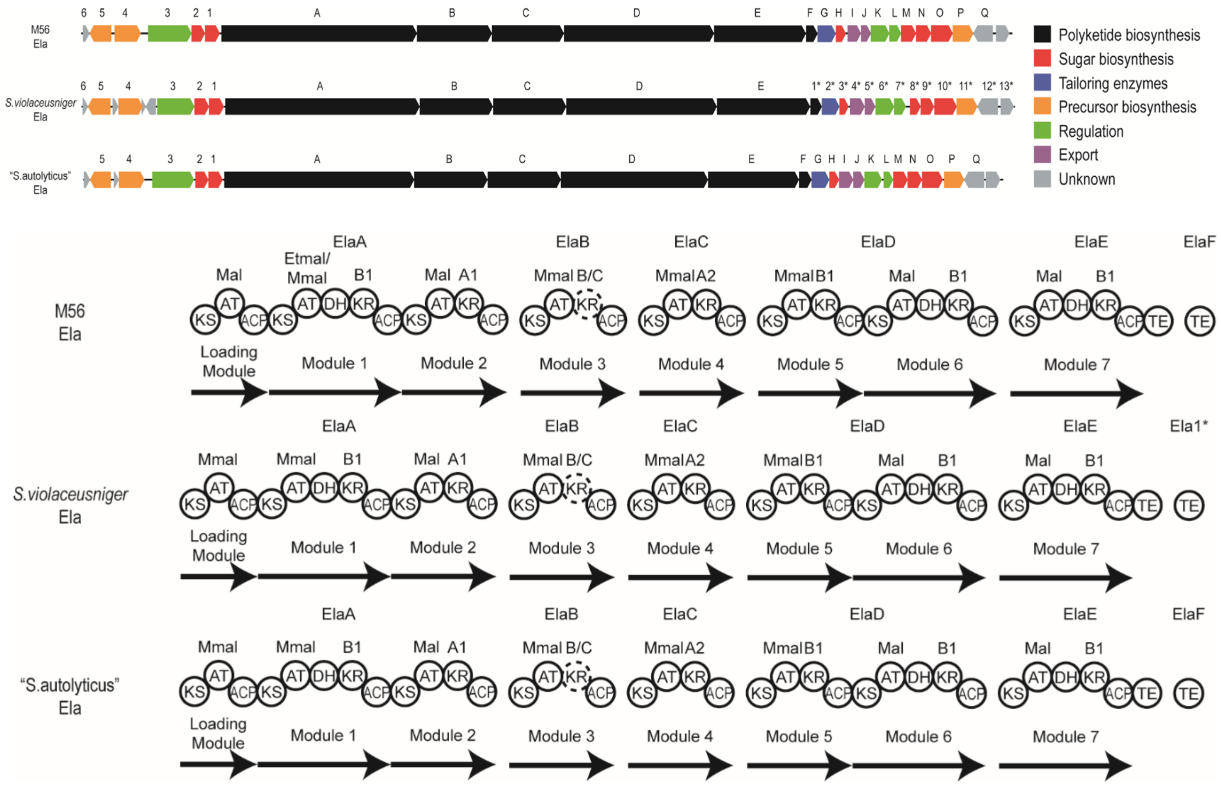

Figure S2. Comparison of *ela* BGCs. Coloured genes are homologous and named following the "*S. autolyticus*" nomenclature and domain composition and specificity of the *ela* BGCs.

Table S2. KR domain specificities for *ela* gene clusters

Tyl6_A1	HTAGTPHSAEF	SSGAAVWGSGGQTAYGAANA
Sor6_A1	HAGGIEPHAPL	SSGAVVWGGGQQGGYAAANA
Ole6_A1	HTAGVPDSRPL	SSNAGVWGSGGQAVYAAANA
Pik5_A1	HTAGAPGGDPL	SSNAGVWGSGWQGVYAAANA
Meg6_A1	HAAGVPQSTPL	SSGAGVWGSANLGAYAAANA
Ery2_A1	HAAGLPQQVAI	SSGAGVWGSARQGAYAAGNA
ElaA2_KR3_Sa	HAAGVVEFSQL	SSIAATWGSGGQSAYAAANA
ElaA_KR3_M56	HAAGVVEFSQL	SSIAATWGSGGQSAYAAANA
ElaA_KR3_Sv	HAAGVVEFSQL	SSIAATWGSGGQSAYAAANA
Can13_A2	HTAAVIELQSI	SSTAGMWGSGRHAAYVAANA
Pim7_A2	HTAVTIELAPL	SSTAGMWGSGAHAAYVAGNA
Ela4_A2	HIAGAGVLVPL	SSISAVWGSGEHGAYAAANA
Amp1_A2	HTAAVIELAAL	SSTAGMWGSGVHAAYVAGNA
Nys1_A2	HAAAAIELSAL	SSTAGMWGSGVHAAYVAGNA
Con5_A2	HAAGTGLLVPL	SSISGVWGSGDHGAYAAANA
ElaC_KR1_Sv	HIAGAGVLVPL	SSISAIWGSGEHGAYAAANA
ElaC_KR1_M56	HIAGAGVLVPL	SSISAVWGSGEHGAYAAANA
ElaC_KR1_Sa	HIAGAGVLVPL	SSISAVWGSGEHGAYAAANA
Tyl1_B1	HTAGILDDAVI	SSAAATFGAPGQANYAAANA
Asc8_B1	HTAATLDDGIL	SSAAAVLGSPGQGNYAAANA
Ave7_B1	HAAGVLDDATI	SSAAGILGSAGQGNYAAANA
Ave9_B1	HAAGVLDDATI	SSAAGILGSAGQGNYAAANA
Rap10_B1	HTAGVLDDGVV	SSAAGVLGSAGQGNYAVANA
Ave1_B1	HTAGILDDATL	SSVTGTWGNAGQGAYAAANA
ElaA_KR2_Sv	HAAGVLDDGVV	SSVVATIGNAGQANYAAANA
ElaA1_KR2_Sa	HAAGVLDDGVV	SSVVATIGNAGQANYAAANA
ElaA_KR2_M56	HAAGVLDDGVV	SSVVATIGNAGQANYAAANA
ElaD_KR1_M56	HTAGVLDDGVL	SSFAGTLGGPGQGSYAAANA
ElaD_KR1_Sa	HTAGVLDDGVL	SSFAGTLGGPGQGSYAAANA
ElaD_KR1_Sv	HTAGILDDGVL	SSFAGTLGGPGQGSYAAANA
ElaD_KR2_M56	HTAGVLDDGVV	SSIAGTFGGMGQGNYAAANA
ElaD_KR2_Sa	HTAGVLDDGVV	SSIAGTFGGMGQGNYAAANA
ElaD_KR2_Sv	HTAGVLDDGVV	SSIAGTFGGMGQGNYAAANA
ElaE_KR1_Sa	HTAGVLDDGVL	SSAAGVLGGAGQGNYAAANG
ElaE_KR1_Sv	HTAGVLDDGVL	SSAAGVLGGAGQGNYAAANG
ElaE_KR1_M56	HTAGVLDDGVL	SSAAGVLGGAGQGNYAAANG
Meg1_B2	HVAATLDDGTV	SSSTAAFGAPGLGGYVPGNA
Ery1_B2	HAAATLDDGTV	SSFASAFGAPGLGGYAPGNA
Lan1_B2	HTAATLDDGTL	SSFASAFGAPGLGCYAPGNA
Pik1_B2	HTAGALDDGIV	SSVSSTLGIPGQGNYAPHNA
ElaB_KR1_Sa	HAAGALDDATV	SSIAGTVGAAGQGNYAAASA
ElaB_KR1_Sv	HAAGALDDATV	SSIAGTVGAAGQGNYAAASA
ElaB_KR1_M56	HAAGALDDATV	SSIAGTVGAAGQGNYAAASA
Oli14_C1	HTAGVAGHGPL	SSGAAVWGSGSNGANAAAGG
Meg3_C2	HAETLTNFAGV	SSVAGVWGGVGMAAYAAGSA
Ery3_C2	HAGTLTNFGSI	SSVAGIWGGAGMAAYAAGSA
Lan3_C2	HAATRTEFGPV	SSVAGVWGGAGMAGYAAGSA
Pik3_C2	HLPPTVDSEPL	SSVAAIWGGAGQGAYAAGTA
Nid4_C2	HAPPLVPLAPL	SSVSGVWGGAAQGAYAAATA
Tyl4_C2	VAPPAVPPTPL	SSVAGVWGGAGQGGYAAGTA

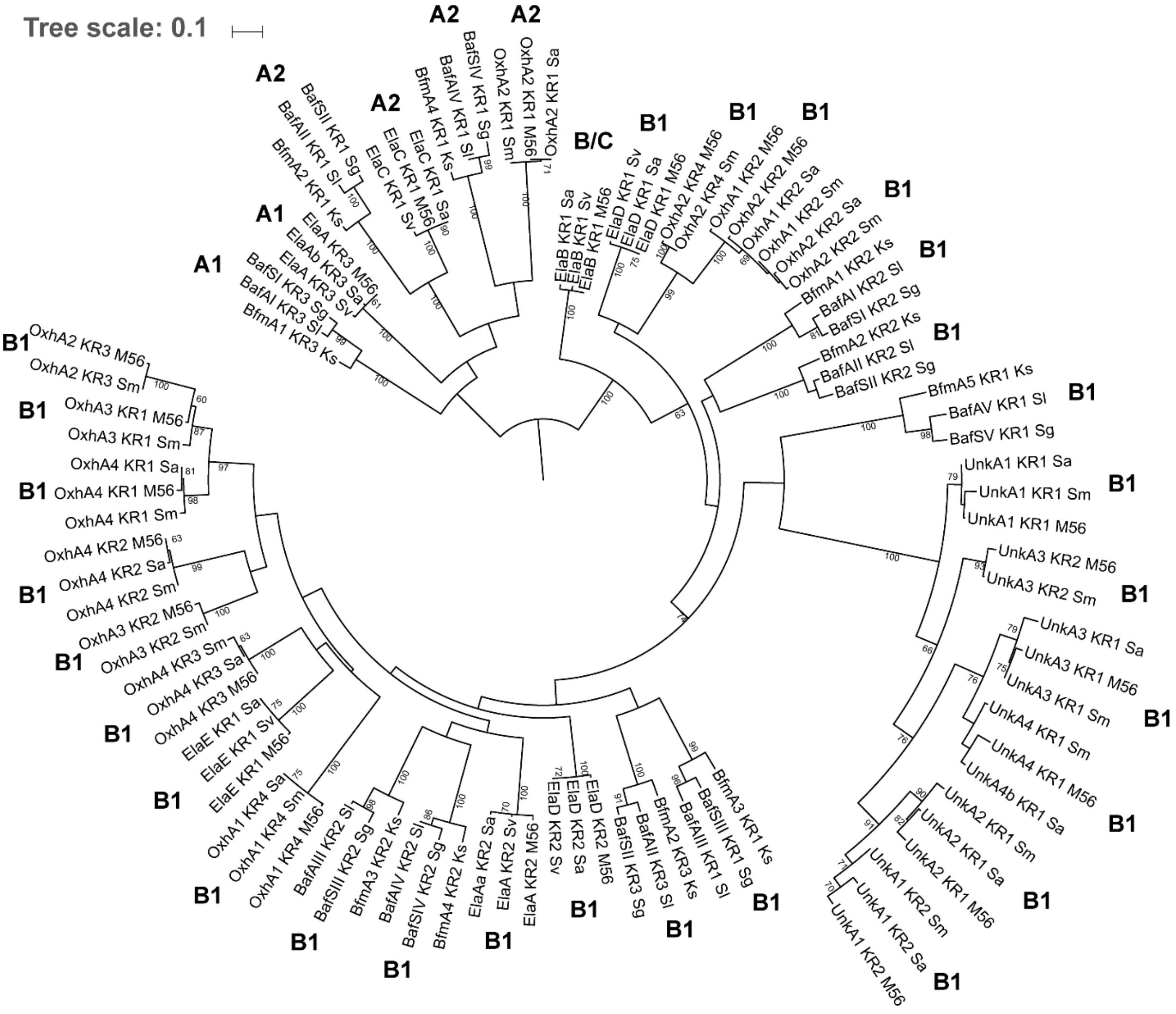

Figure S3. Phylogenetic analysis of KR domains of type 1 PKS-related biosynthetic gene clusters found in M56 (*ela*, *oxh*, *baf*/*bfm*, and *unk*). Protein sequences were aligned using MUSCLE v3.8.31 using default parameters, and approximate maximum likelihood trees were constructed using RAxMLv8.0.26 with automatic model selection and 1000 bootstrap pseudo-replicates. Only bootstrap values ≥ 60% are shown. The tree was rooted to an arbitrarily long branch for clarity, and letters to the right of each set of orthologous domains indicate their domain specificity.

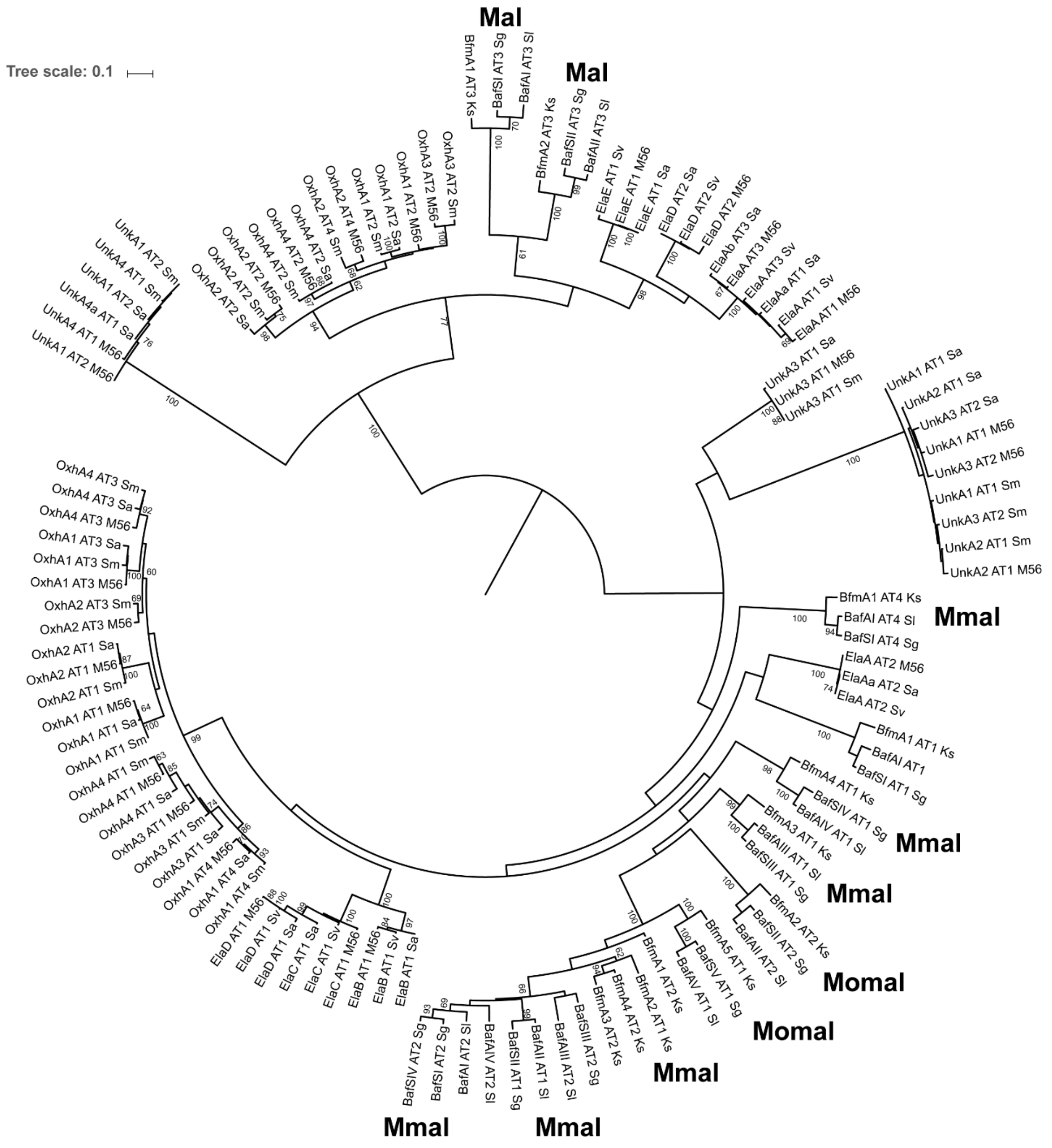

Figure S4. Phylogenetic analysis AT domain type 1 PKS-related biosynthetic gene clusters found in M56 (*ela*, *oxh*, *baf*/*bfm*, and *unk*). Protein sequences were aligned using MUSCLE v3.8.31 using default parameters, and approximate maximum likelihood trees were constructed using RAxMLv8.0.26 using automatic model selection and 1000 bootstrap pseudo-replicates. Only bootstrap values ≥ 60% are shown. The tree was rooted to an arbitrarily long branch for clarity, and the domain specificities assigned previously to the Baf/Bfm AT domains are indicated beside each clade.

3. Metabolomic Analysis

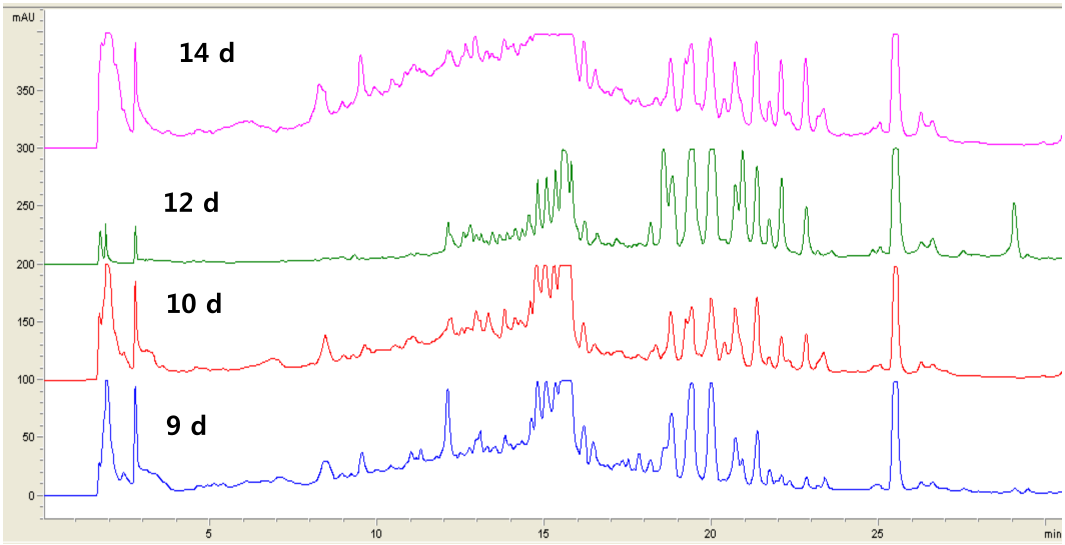

Figure S5. Adapted LC-MS diagram (254 nm) of metabolite extracts from *Streptomyces* sp. M56 grown on ISP-2 agar plates after 9 d (blue), 10 d (red), 12 d (green), and 14 d (pink).

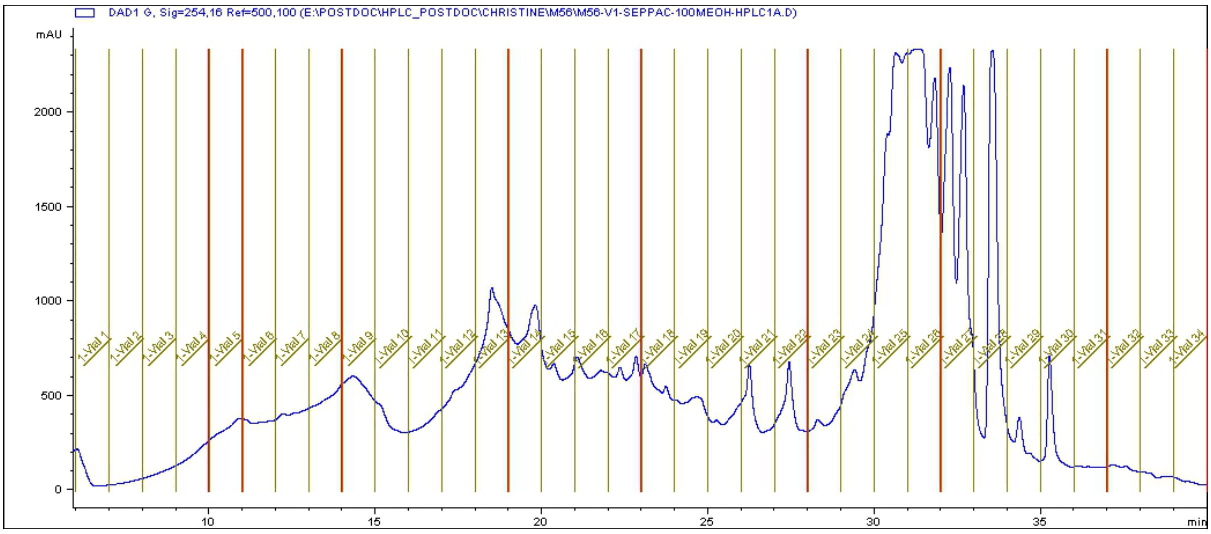

Figure S6. Preparative HPLC chromatogram (254 nm) of metabolite extracts from *Streptomyces* sp. M56 grown on ISP-2 agar plates.

4. Structure Analysis and Analytical Data

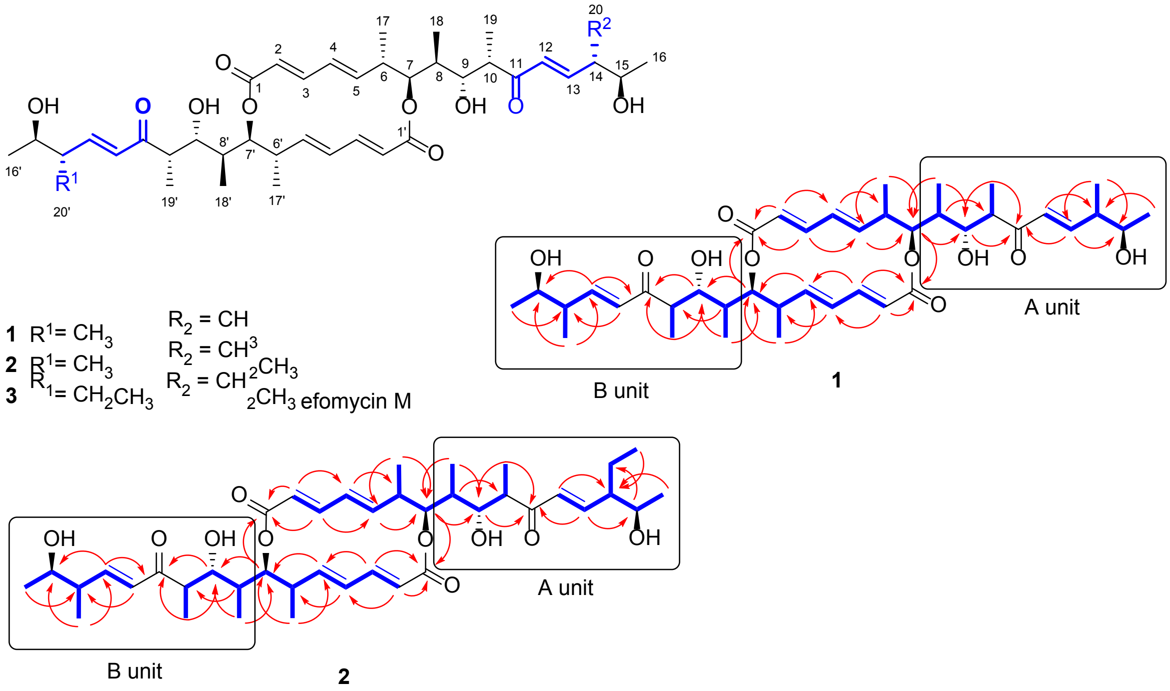

Figure S7. Key ^{1}H-^{1}H COSY and TOCSY (blue lines) and HMBC (red arrows) correlations for compounds **1** and **2**.

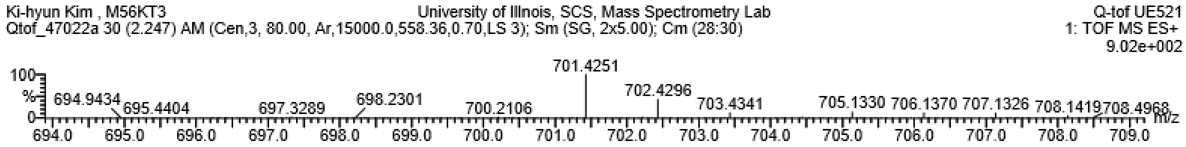

Minimum:				-1.5		
Maximum:		5.0	10.0	600.0		
Mass	Calc. Mass	mDa	PPM	DBE	i-FIT	Formula
701.4251	701.4265	-1.4	-2.0	10.5	0.5	C40 H61 O10

Figure S8. HR-ESI-MS data of **1**.

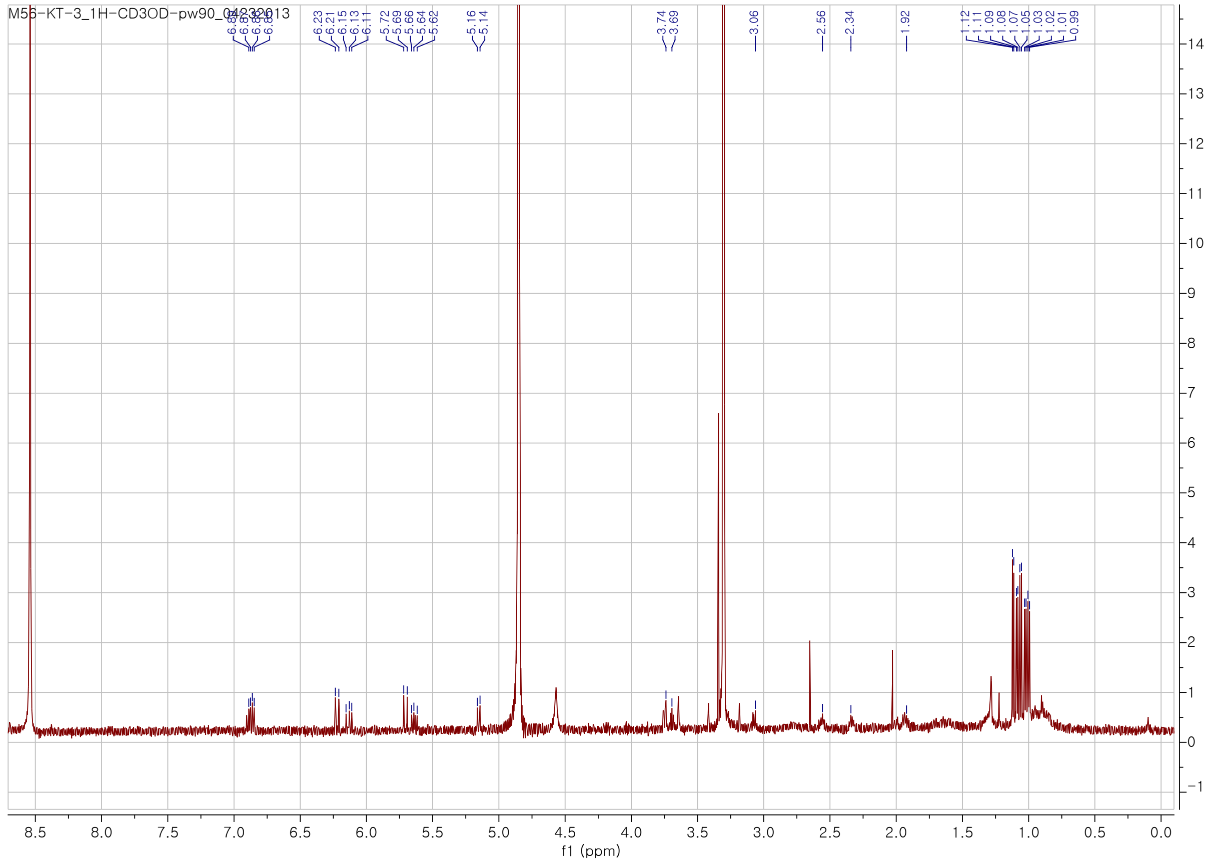

Figure S9. ^{1}H NMR spectrum of **1**.

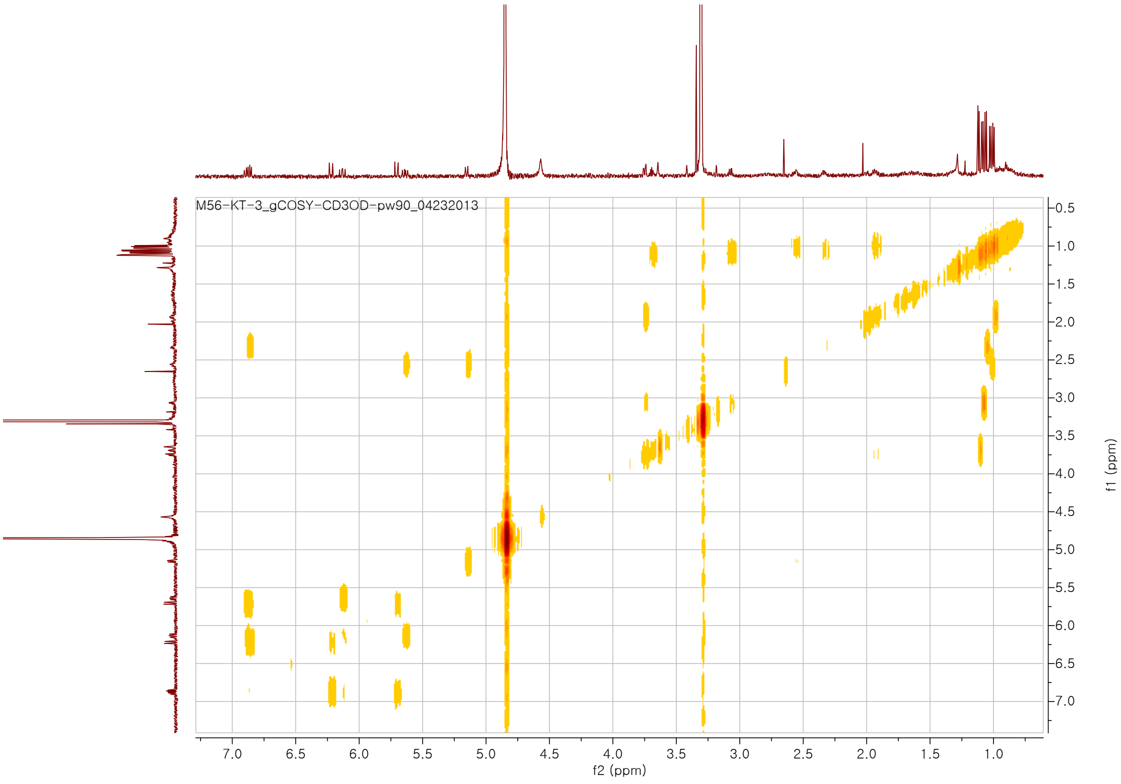

Figure S10. ^{1}H-^{1}H COSY spectrum of **1.**

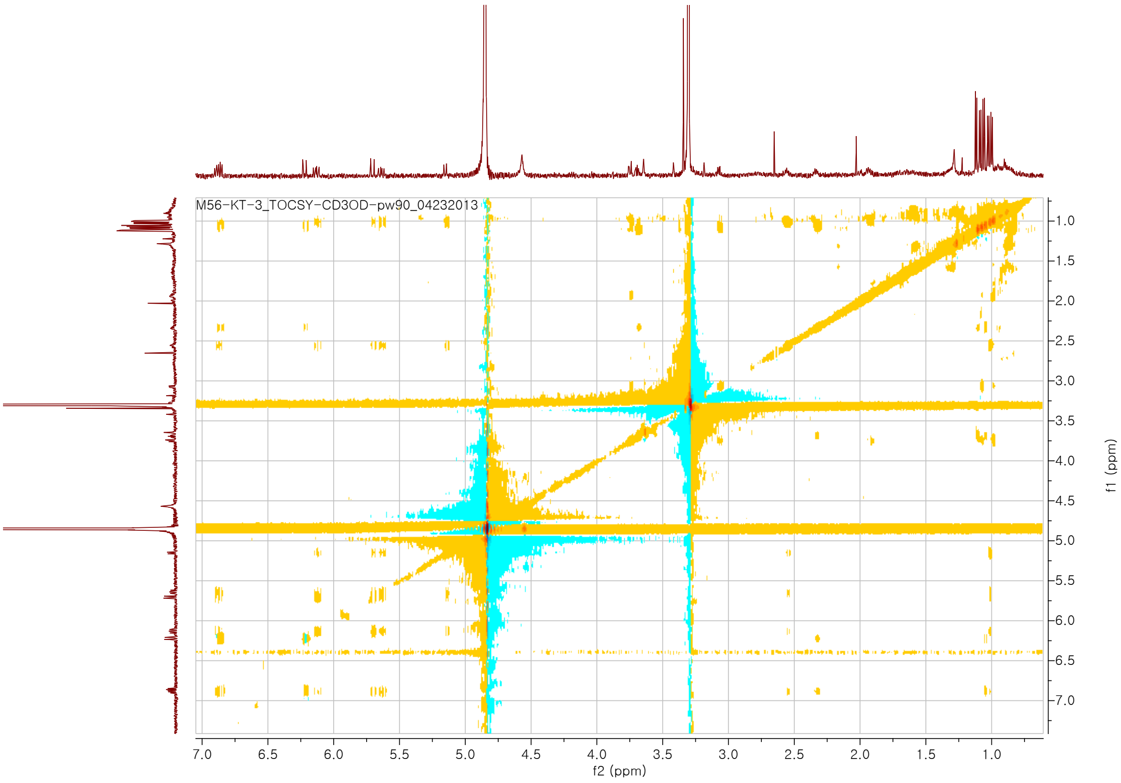

Figure S11. TOCSY spectrum of **1.**

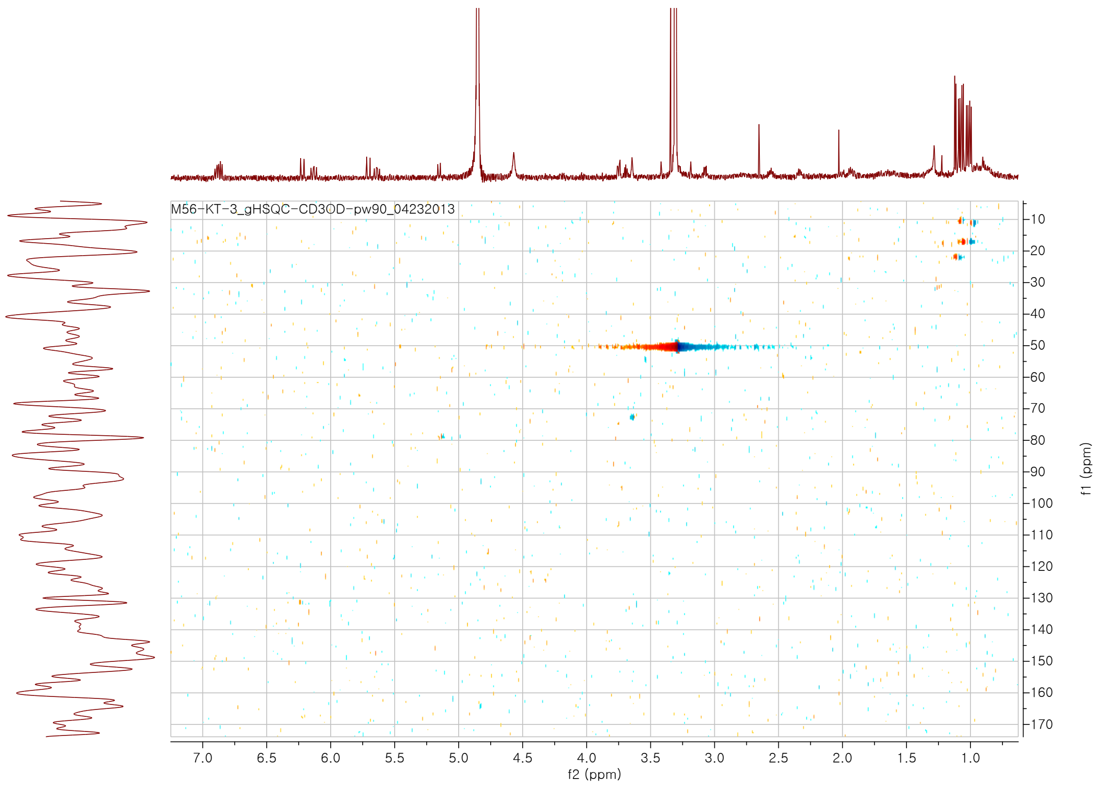

Figure S12. HSQC spectrum of **1.**

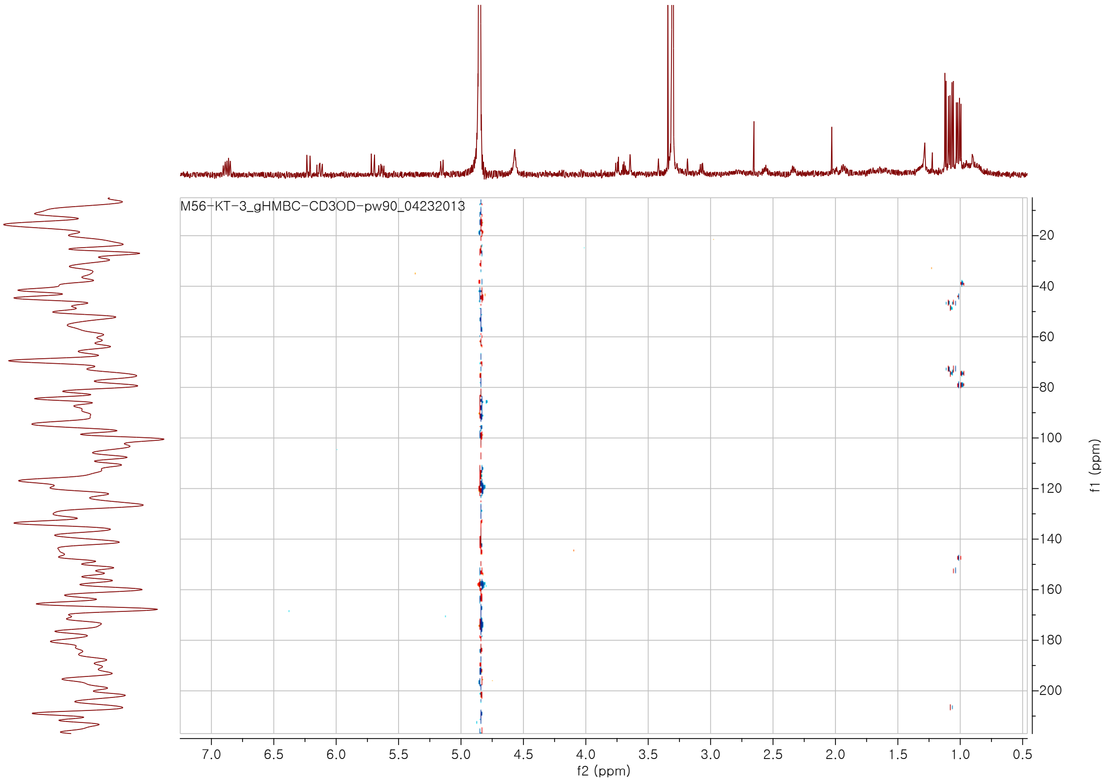

Figure S13. HMBC spectrum of **1.**

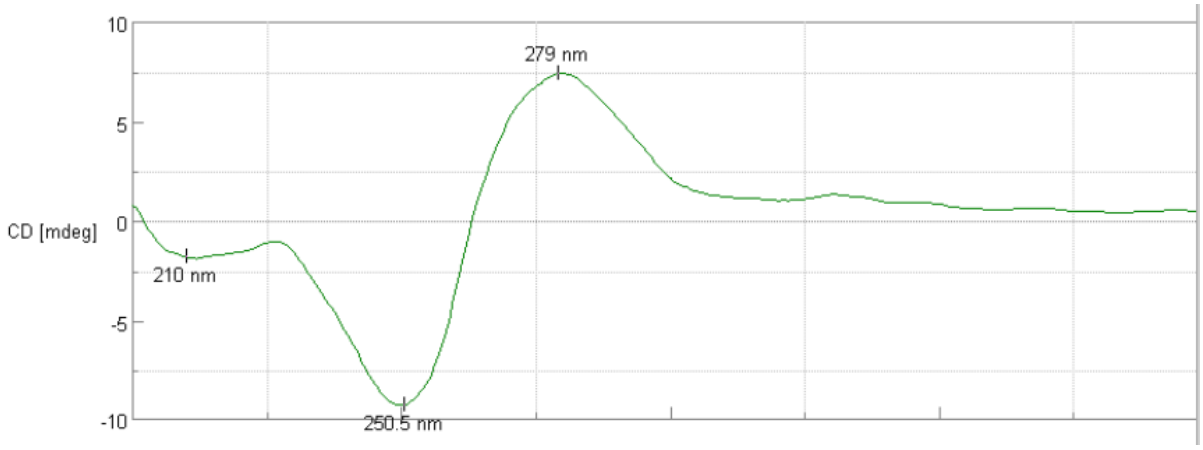

Figure S14. ECD spectrum of **1.**

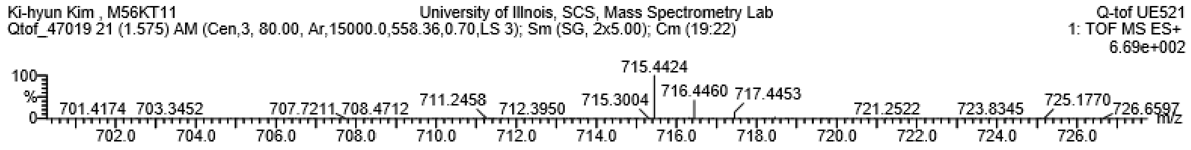

Minimum:				-1.5		
Maximum:		5.0	10.0	600.0		
Mass	Calc. Mass	mDa	PPM	DBE	i-FIT	Formula
715.4424	715.4421	0.3	0.4	10.5	2.1	C41 H63 O10

Figure S15. HR-ESI-MS data of **2.**

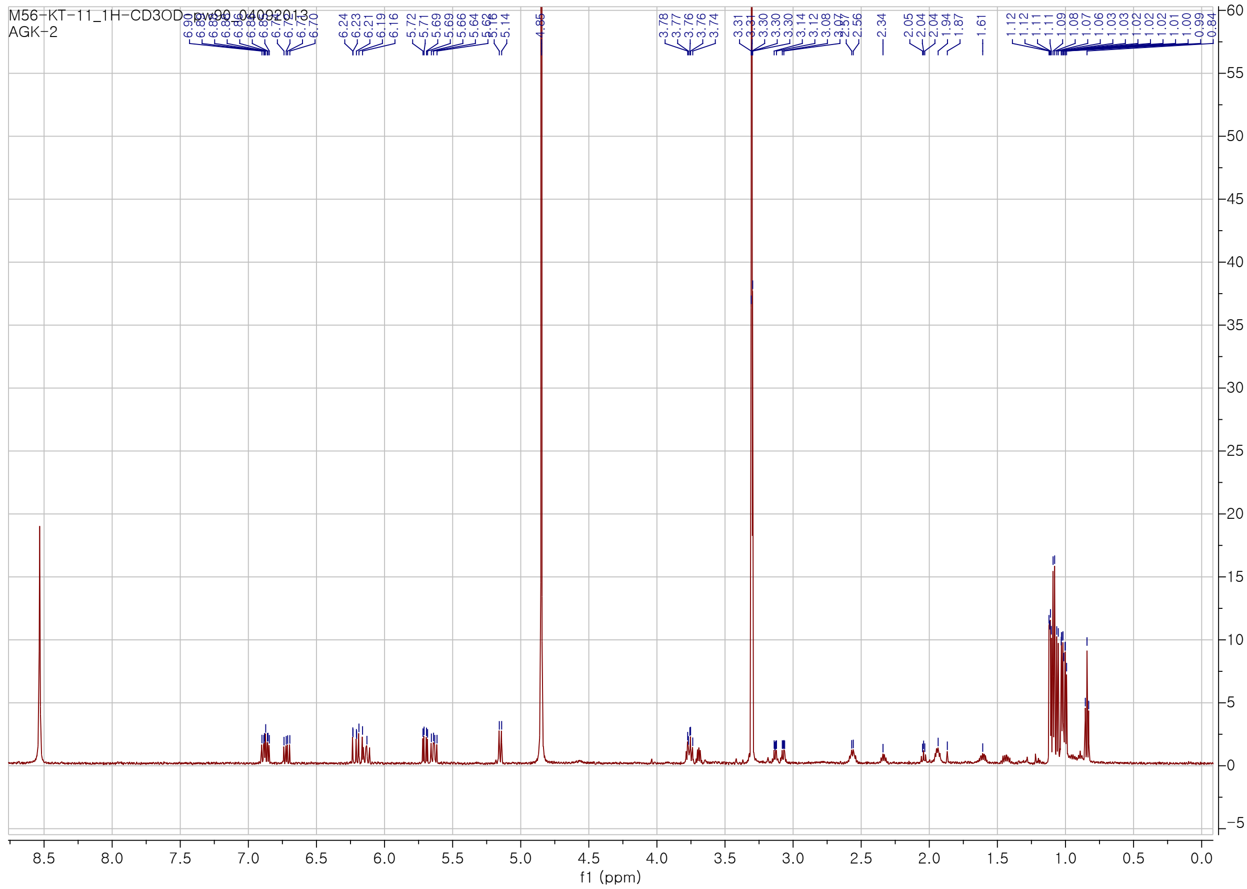

Figure S16. ^{1}H NMR spectrum of **2.**

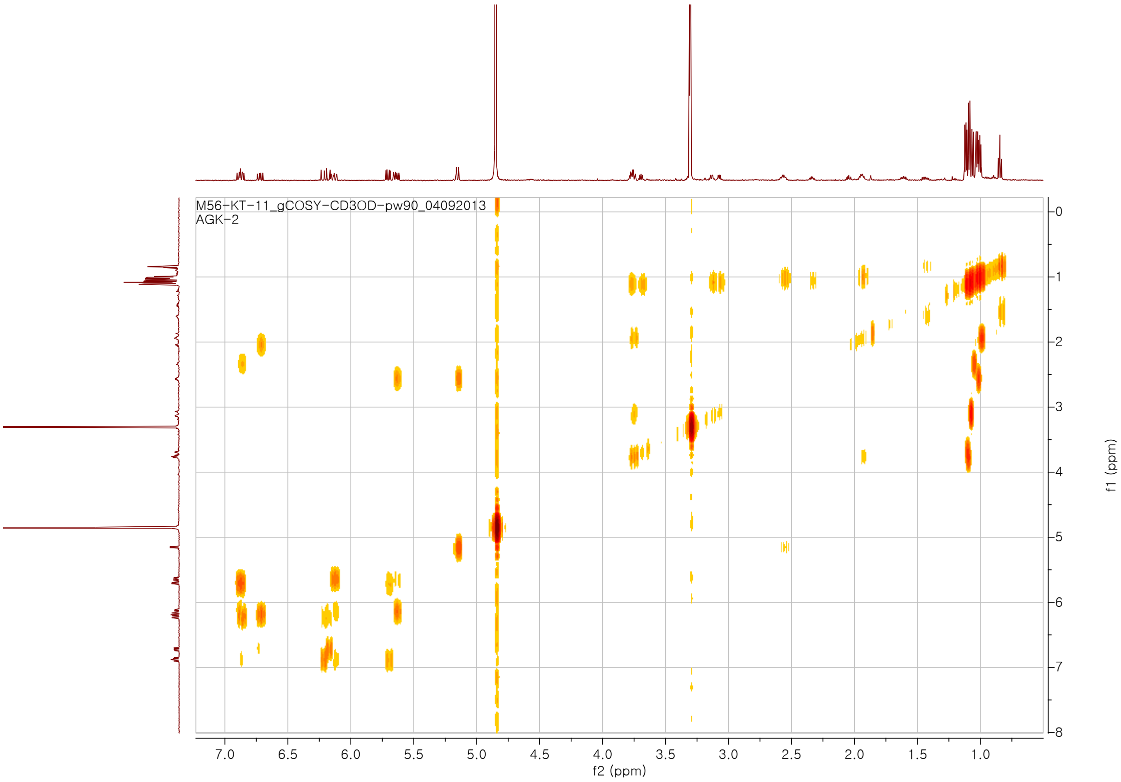

Figure S17. ^{1}H-^{1}H COSY spectrum of **2.**

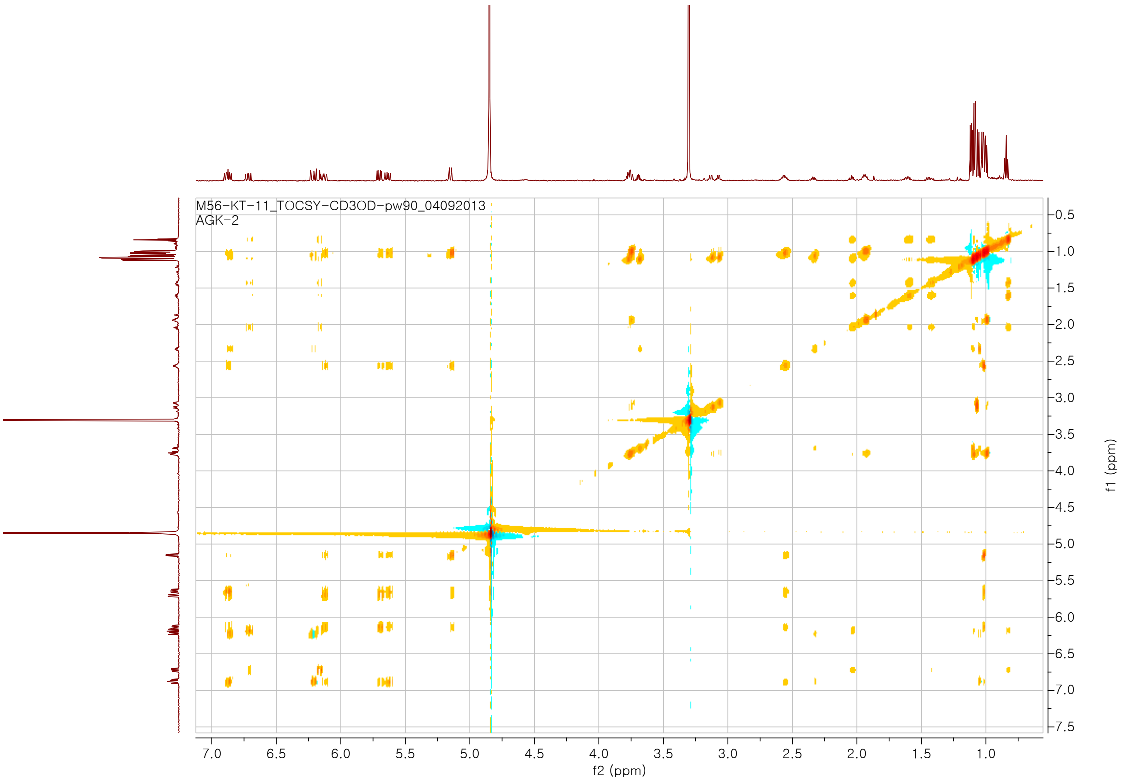

Figure S18. TOCSY spectrum of **2.**

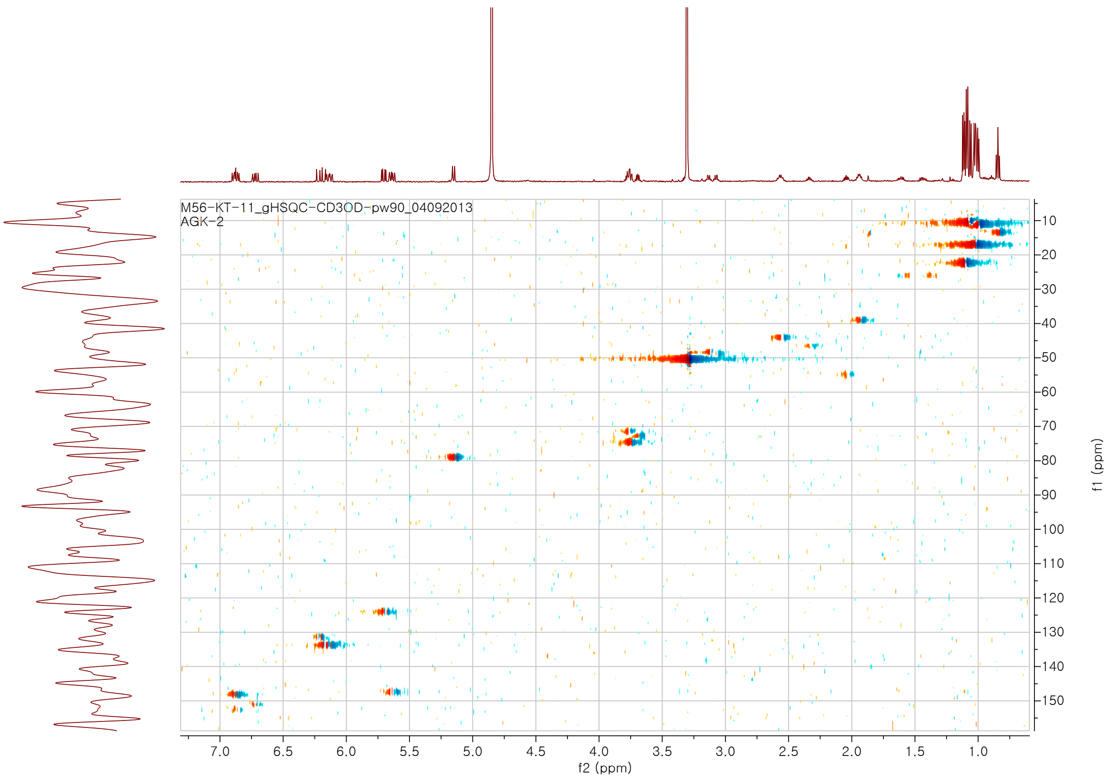

Figure S19. HSQC spectrum of **2.**

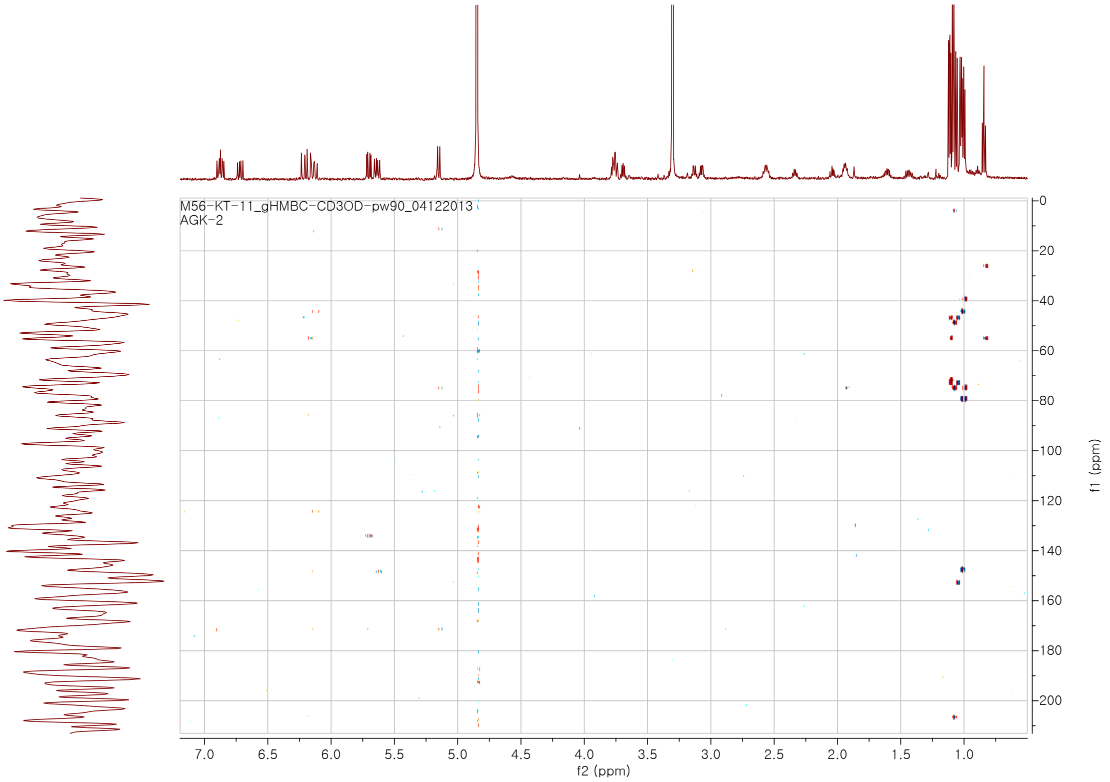

Figure S20. HMBC spectrum of **2**

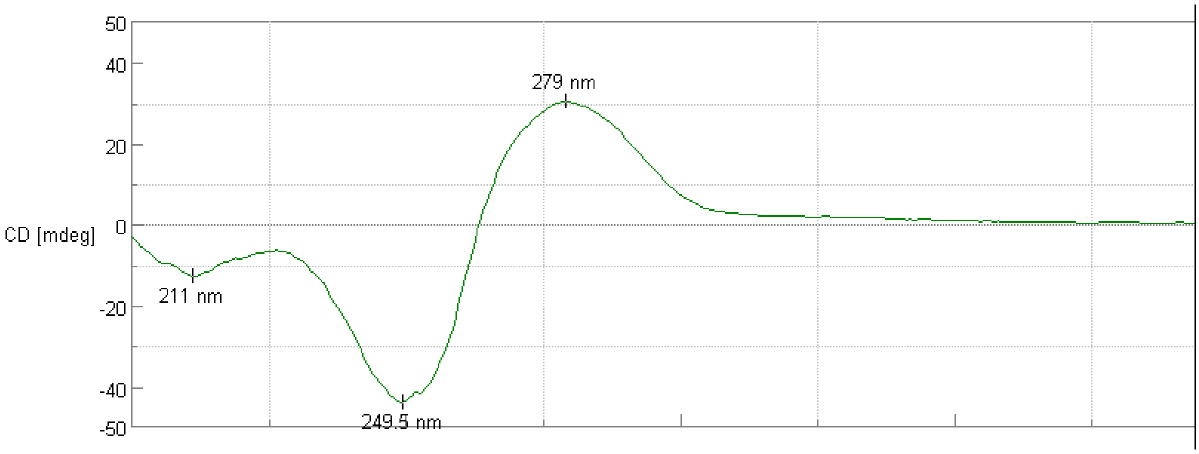

Figure S21. ECD spectrum of **2.**
